# Changes in Oxidative Stress, Inflammatory and Bone Metabolism Biomarkers Following Sulfurous Water Inhalation in Osteopenic Women

**DOI:** 10.3390/ijms27073163

**Published:** 2026-03-31

**Authors:** Laura Gambari, Emanuela Amore, Livia Roseti, Sara Carpentieri, Claudio Ripamonti, Lucia Lisi, Paolo Spinnato, Giuliana Nervuti, Antonietta Gesuele, Susanna Naldi, Brunella Grigolo, Francesco Grassi

**Affiliations:** 1 SSD Lab RAMSES, IRCCS Istituto Ortopedico Rizzoli, Via di Barbiano 1/10, 40136 Bologna, Italy; laura.gambari@ior.it (L.G.); emanuela.amore@ior.it (E.A.); livia.roseti@ior.it (L.R.); sara.carpentieri@ior.it (S.C.); brunella.grigolo@ior.it (B.G.); 2 SSD Medicina e Reumatologia, IRCCS Istituto Ortopedico Rizzoli, Via di Barbiano 1/10, 40136 Bologna, Italy; claudio.ripamonti@ior.it (C.R.); lucia.lisi@ior.it (L.L.); susanna.naldi@ior.it (S.N.); 3 SC Radiologia Diagnostica e Interventistica, IRCCS Istituto Ortopedico Rizzoli, Via di Barbiano 1/10, 40136 Bologna, Italy; paolo.spinnato@ior.it; 4 SC Chirurgia Vertebrale, IRCCS Istituto Ortopedico Rizzoli, Via di Barbiano 1/10, 40136 Bologna, Italy; giuliana.nervuti@ior.it; 5 SC SAIteR, IRCCS Istituto Ortopedico Rizzoli, Via di Barbiano 1/10, 40136 Bologna, Italy; antonietta.gesuele@ior.it

**Keywords:** osteoporosis, menopause, hydrogen sulfide, clinical study, sulfurous waters, oxidative stress, inflammation

## Abstract

Postmenopausal osteoporosis is an age-related condition in which estrogen deficiency drives low-grade inflammation and oxidative stress, disrupting the homeostatic balance between bone formation and resorption. Since osteopenia represents a critical intermediate stage, preventive strategies are essential to mitigate its progression. Preclinical studies suggest that hydrogen sulfide (H_2_S), a gaseous mediator with antioxidant properties, protects bone metabolism by supporting osteoblast function and suppressing osteoclast activity. Building on this evidence, we conducted the first exploratory clinical trial assessing the effects of inhalation therapy with sulfurous mineral waters on systemic biomarkers in postmenopausal women with osteopenia. Thirty-eight eligible participants underwent a daily inhalation of sulfurous waters (14.6 mg/L sulfide) for 12 consecutive days. Biomarkers of oxidative stress, inflammation, and bone turnover were assessed at baseline, immediately post-treatment, and five days after cessation in the serum of patients. The treatment was well tolerated and did not cause any early adverse effect. Serum H_2_S levels, measured in a subset of participants, significantly increased, confirming systemic bioavailability. Sulfurous water inhalation induced a marked change in oxidative stress, with malondialdehyde levels declining by up to 37% from baseline. Pro-inflammatory cytokines, particularly IL-8 and MIP-1α, were significantly decreased (up to 50–70%) at the end of the treatment. Reference bone turnover markers P1NP and CTX-1 did not show significant changes; however, BALP exhibited a significant increase, suggesting the activation of pathways linked to biomineralization. These findings provide preliminary human evidence that inhaled sulfurous waters enhance systemic H_2_S bioavailability and modulate redox and inflammatory pathways associated with bone remodeling in osteopenic women, supporting the rationale for further controlled pharmacodynamic investigations evaluating the potential of H_2_S in bone health.

## 1. Introduction

Osteoporosis is a major public health concern characterized by diminished bone density, deteriorated bone microarchitecture and increased susceptibility to fractures [1]. According to the World Health Organization, osteoporosis is defined as a bone mineral density (BMD) T score of −2.5 or lower and osteopenia as a T score between −1.0 and −2.5 [2,3], as measured by Dual-energy X-ray absorptiometry (DXA); based on this diagnostic criteria, the global prevalence of osteoporosis and osteopenia was estimated, respectively, to be 19.7% and 40.4% [4].

The progressive loss of bone mass is typical of aging, and, in women, it is strongly accelerated by estrogen deficiency during menopause [5]. This is consistent with the evidence that estrogen is the dominant sex hormone regulating bone metabolism [6,7]. The prevalence of osteoporosis and osteopenia was, respectively, 27.4% and 42.1% in postmenopausal women [4].

Osteopenia is a condition of intermediate risk [8] characterized by a lower risk of bone fracture compared to osteoporosis. However, clinical evidence suggests that a significant share of osteopenic women will transition to osteoporosis within a few years [9]; moreover, although individuals with osteoporosis are at a higher fracture risk than those with osteopenia, most fractures occur in the larger osteopenic population, suggesting that targeting this currently untreated group could have a greater impact on overall fracture rates [10,11,12]. While pharmacological treatments are critical for managing postmenopausal osteoporosis, non-pharmacological approaches are equally important for its prevention and for promoting overall bone health, particularly among osteopenic women [11,12].

In the condition of estrogen deficiency, one important driver of the imbalance between bone resorption and bone formation is the rise in subclinical inflammation linked to an increased production of pro-inflammatory cytokines by specific T cell subsets, which promote pro-resorptive and anti-osteogenic effects in bone [13,14,15,16]; in women, increased levels of inflammatory biomarkers were observed as early as 1 year before the final menstrual period [17]. Moreover, the onset of menopause has been associated, in both preclinical and clinical studies, with an increased production of reactive oxygen species (ROS) [18,19,20] which accumulate at concentrations higher than those required for normal cell functioning even in bone cells [21]. Consistent with preclinical findings, it was reported that postmenopausal osteoporotic women have higher levels of circulating advanced oxidation protein products (AOPPs) and malondialdehyde (MDA) compared with age-matched controls [22], and that oxidative stress is an independent risk factor for osteoporosis [13,23]. Furthermore, a clinical study found that higher levels of serum 8-hydroxy-2′-deoxyguanosine (8-OH-dG, a marker of oxidative stress) were inversely correlated with BMD and positively associated with bone resorption markers in postmenopausal women [24]. Physiological bone remodeling and bone repair are indeed redox-regulated processes; ROS work as essential intracellular signaling molecules in osteoclast (OC) differentiation while their suppression halts OC development entirely [25,26]. On the other hand, osteoblasts are harmed by excessive ROS in multiple ways: decreased osteoprogenitor differentiation [27], a decreased production of osteoprotegerin (OPG) [28] and increased osteoblast and osteocyte apoptosis were demonstrated in vitro and in vivo under conditions of excess ROS signaling. As further evidence of the importance of oxidative stress, various approaches aimed at reducing it have long been proposed as a strategy for mitigating bone loss and restoring a balance between bone formation and bone resorption in populations at risk [21].

Hydrogen sulfide (H_2_S) is a pleiotropic gaseous molecule produced endogenously by enzymes involved in the so-called transsulfuration pathway [29,30], which plays various essential roles in organs and tissues [31,32,33,34]. In bone, H_2_S has been shown to act as a multi-dimensional regulator of bone cell metabolism by maintaining the pool of osteoprogenitor stem cells [35]; protecting osteoblastic cells against oxidative stress-induced cell injury [36]; promoting osteoblast differentiation in vitro [35,37]; and protecting against ovariectomy-induced bone loss in mice by stimulating bone formation in vivo [37]. Moreover, our group and others showed that H_2_S strongly suppresses the formation and activity of human osteoclasts in vitro [38,39,40] by inducing the nuclear translocation of NRF2, and activating antioxidant genes (e.g., NQO1, PRDX1) that reduce ROS. To translate preclinical evidence of H_2_S’s osteoprotective effects into a clinical setting, we utilized sulfurous mineral waters as a natural and validated source for H_2_S inhalation therapy.

Sulfurous water inhalation has a longstanding tradition in the management of airway disorders, including laryngitis, pharyngitis, and rhinitis [41,42]. More recently, its therapeutic application has been extended to chronic obstructive pulmonary disease (COPD) and COVID-19, primarily due to its demonstrated anti-inflammatory and antioxidant properties in clinical research trials [43]. Based on these preliminary findings, we hypothesized that the exogenous administration of an aerosol enriched with H_2_S may combine anti-inflammatory and antioxidant effects, thereby mitigating the detrimental effect of menopause on bone in osteopenic women.

To investigate this hypothesis, we conducted the first single-arm exploratory clinical trial among postmenopausal women at risk of developing osteoporosis, using sulfurous mineral waters with a known concentration of sulfide as a carrier of H_2_S for 12 days, following a standard clinical protocol used in thermal medicine. The levels of circulating biomarkers of inflammation, oxidative stress and bone turnover were evaluated before and after treatment.

## 2. Results

**Figure 1:**
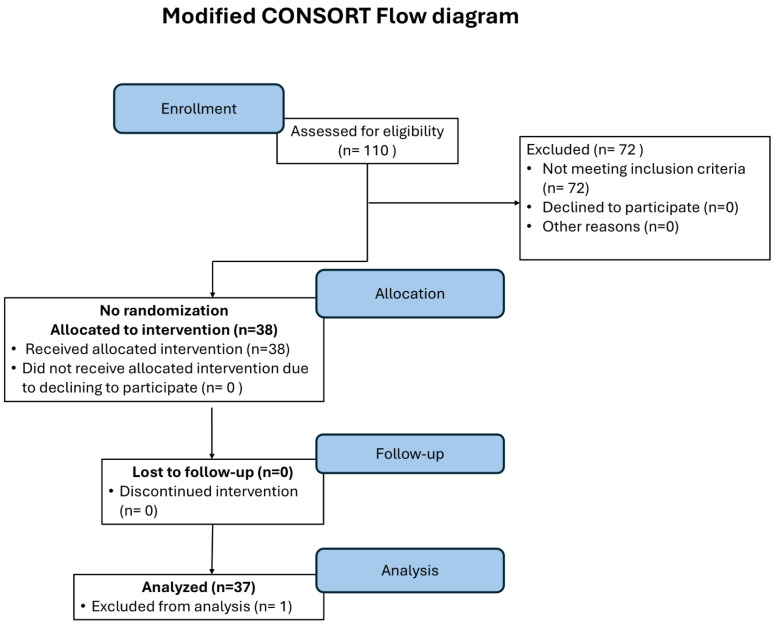
Modified Consort diagram of the clinical trial illustrating the experimental design and the study population.

The diagram flow of the single-arm exploratory clinical trial is depicted in Figure 1. A total of 110 women were initially assessed for eligibility. After preliminary interview, a group of 33 women were excluded for not meeting the inclusion criteria and five declined to participate; the remaining 72 patients were screened for BMD by performing a DXA scan after signing the informed consent. Based on the outcome of the DXA, 34 more women were excluded from the study for not meeting the inclusion criteria of a BMD in the range of osteopenia. Therefore, at the end of the screening phase, 38 women (35.2%) were allocated to the inhalatory treatment.

No early adverse events were reported during the treatment. After the end of treatment, one patient was excluded from analysis as she experienced a return of menstrual period.

### 2.1. Measurement of Serum H_2_S Levels

**Figure 2:**
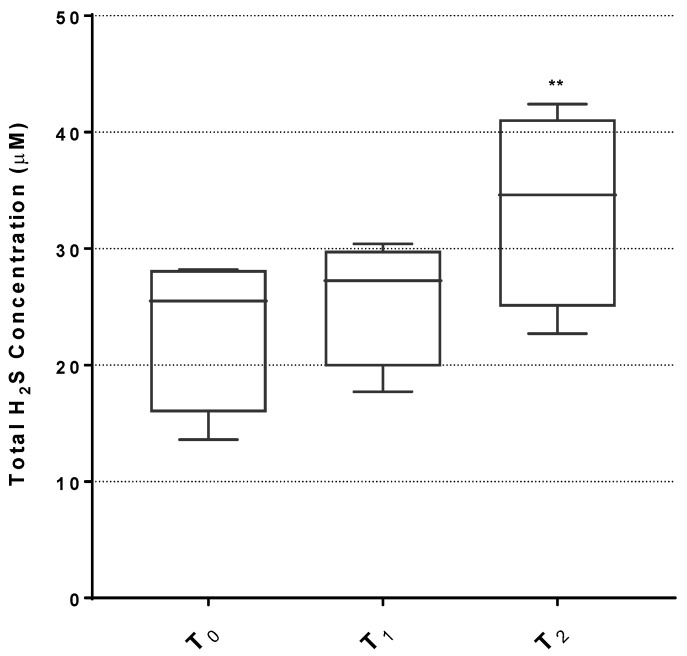
Total H_2_S levels in the serum of women before and after treatment. Box plots represent the median and the 10–90th percentile of serum H_2_S levels (n = 4). ** = *p* < 0.01 vs. T_0_. T_0_ = baseline; T_1_ = end of treatment; T_2_ = 5-day follow-up after end of treatment.

To evaluate whether the inhalation with sulfurous waters increases the levels of H_2_S in the bloodstream, we undertook a measurement of serum H_2_S on a subset of four patients randomly selected, using a protocol previously published by our group [44]. As shown in Figure 2, the level of total H_2_S is increased at the end of treatment (T_1_) and further increases at the follow-up, 5 days after the cessation of treatment (T_2_), achieving a statistically significant 40% increase (*p* < 0.01) compared to the baseline (T_0_). These findings show that inhalation treatment with sulfurous waters results in increased levels of circulating H_2_S.

### 2.2. MDA Determination

**Figure 3:**
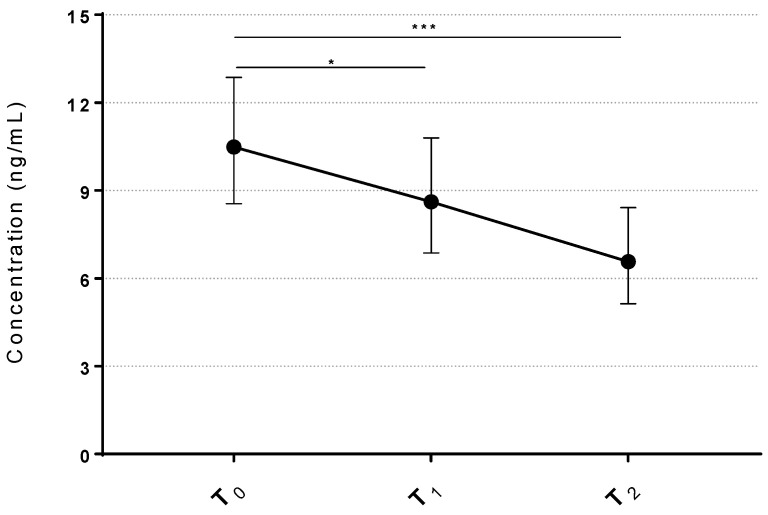
Inhalation of sulfurous waters reduces MDA levels in osteopenic women. Line graph represents statistically significant differences in serum MDA levels before and after the treatment. Data represents the mean ± 95% CI of serum H_2_S levels (n = 37) *** = *p* < 0.001, * *p* < 0.05 vs. T_0_. T_0_ = baseline; T_1_ = end of treatment; T_2_ = 5-day follow-up after end of treatment.

Figure 3 shows the serum concentration of MDA during the time-course of the treatment. The inhalation of sulfurous waters led to a steady and statistically significant decrease in serum MDA concentration among participants (*p* < 0.001), corresponding to a statistically significant decrease of 17.8% at T_1_ (*p* < 0.05) and 37.3% at T_2_ (*p* < 0.001) compared to the baseline levels (T_0_); controlling for potential confounders (age and BMI) did not affect the result of this analysis.

### 2.3. Measurement of Inflammatory Cytokines

**Figure 4:**
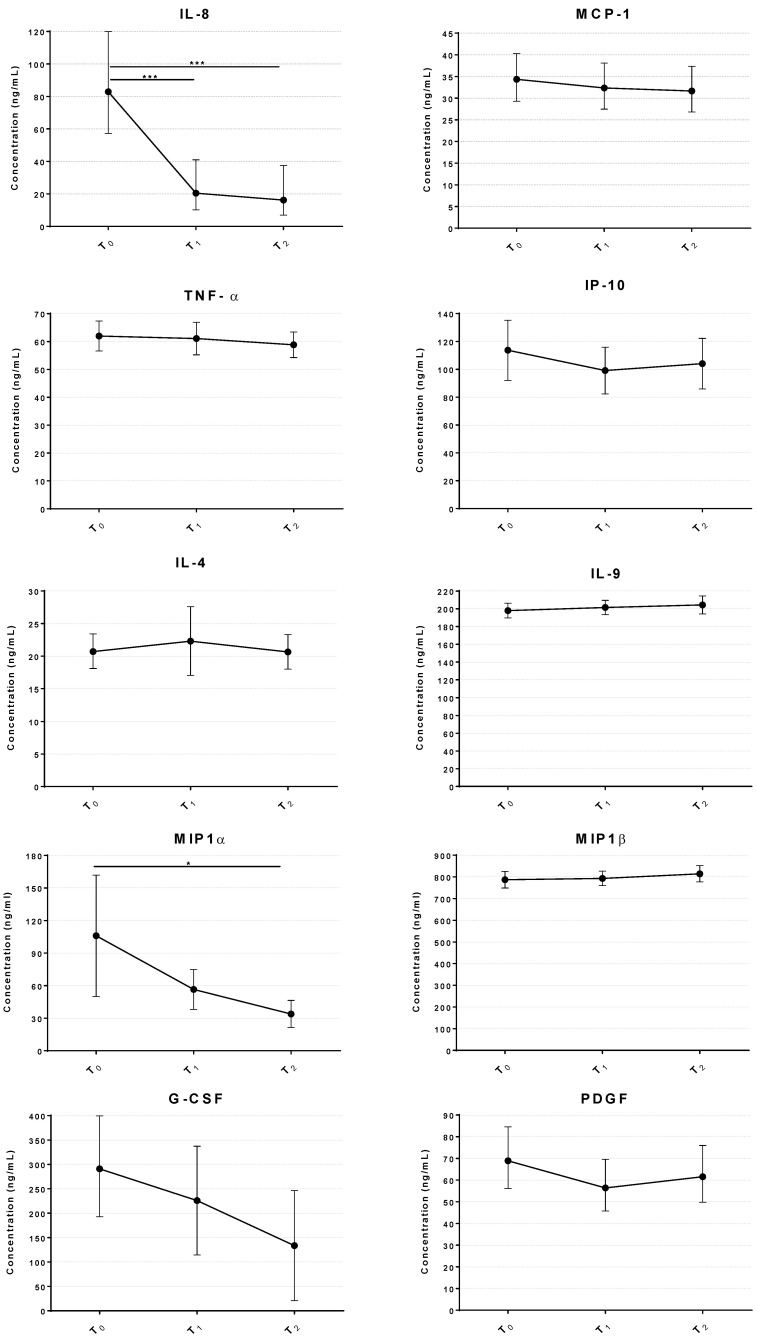
Inhalation of sulfurous waters reduces inflammatory cytokines in osteopenic women. Line graph represents concentration of inflammatory cytokines before and after the treatment. Data represents the mean ± 95% CI of serum cytokine levels (n = 37) *** = *p* < 0.001. * = *p* < 0.05 vs. T_0_. T_0_ = baseline; T_1_ = end of treatment; T_2_ = 5-day follow-up after end of treatment.

To investigate if the sulfurous water inhalation induced a shift in the profile of circulating inflammatory cytokines, we assessed the serum concentrations of the most common pro-inflammatory cytokines before and after treatment. Figure 4 summarizes the results of the 10 cytokines which provided detectable concentrations in patients across the timeline of the treatment. The levels of interleukin-8 (IL-8), monocyte chemoattractant protein 1 MCP-1, tumor necrosis factor α (TNFα), interferon gamma-induced protein (IP-10), macrophage inflammatory proteins 1α (MIP1α), granulocyte macrophage colony-stimulating factor (G-CSF) and platelet-derived growth factor (PDGF) were lower after the treatment compared to the baseline levels; while IL-4, MIP1β and IL-9 were slightly increased. Sulfurous water inhalation caused the levels of IL-8, MIP1α and G-CSF to fall between 50 and 70% relative to baseline. When analyzed though a generalized linear mixed model (GLMM) approach for non-parametric data, we found that changes were highly statistically significant for IL-8 (at both T_1_ and T_2_ *p* < 0.001) and MIP1α (at T_2_ *p* < 0.05) but did not reach significance for G-CSF (*p* = 0.109). Controlling for potential confounders (age and BMI) did not affect the result of this analysis.

### 2.4. Measurement of Vitamin D (VITD) and Parathyroid Hormone (PTH)

**Figure 5:**
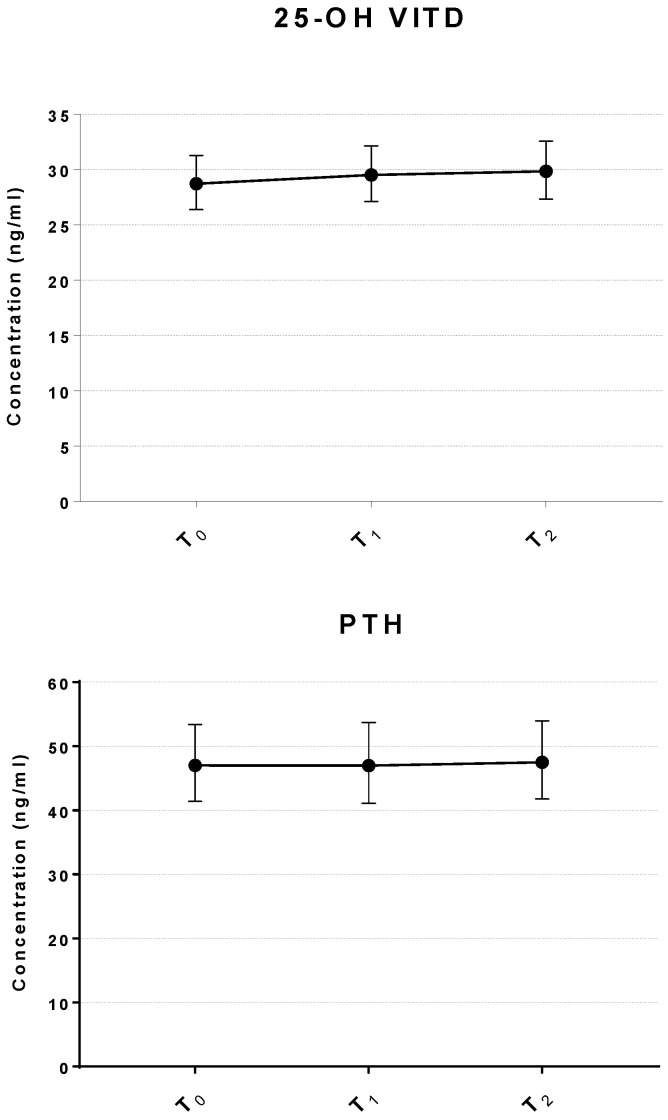
Change in the levels of Vitamin D and PTH following inhalation of sulfurous water in osteopenic women. Line graphs show the serum concentrations of VIT D and PTH before and after the treatment. Data represents the mean ± 95% CI of serum levels (n = 37). T_0_ = baseline; T_1_ = end of treatment; T_2_ = 5-day follow-up after end of treatment.

To investigate if inhalation treatment with sulfurous water changed the concentrations of hormonal regulators of bone metabolism, the levels of VITD and PTH were measured. As shown in Figure 5, the levels of PTH were unchanged after treatment; similarly, the concentrations of VITD increased slightly (3.8% relative to baseline) but the change was not statistically significant (*p* = 0.257). Controlling for potential confounders (age and BMI) did not affect the result of this analysis.

### 2.5. Measurement of Biomarkers of Bone Turnover

**Figure 6:**
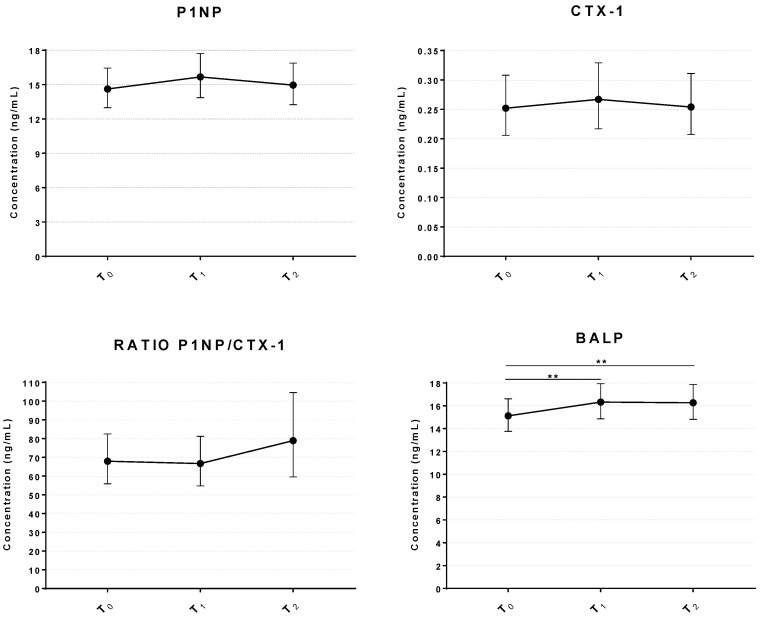
Change in the levels of BTMs following inhalation of sulfurous water in osteopenic women. Line graph shows the serum concentrations of P1NP, CTX and BALP before and after the treatment; the ratio P1NP/CTX-1 is also reported. Data represents the mean ± 95% CI of serum levels (n = 37). ** *p* < 0.01. T_0_ = baseline; T_1_ = end of treatment; T_2_ = 5-day follow-up after end of treatment.

To gain insights into whether the inhalation of sulfurous water could affect bone metabolism, levels of bone turnover markers (BTMs) were assessed before and after treatment. Firstly, we assessed the levels of the two most recommended BTMs for evaluating the response to osteoporosis treatment, Procollagen type 1 N-terminal propeptide (P1NP) and C-terminal telopeptide of type I collagen (CTX-1). As shown in Figure 6, the serum concentrations of P1NP and CTX-1 showed a transient but not statistically significant increase at T_1_, of 7.3% and 5.9% respectively, compared to the baseline; both markers returned to near baseline levels a few days after the cessation of the treatment (T_2_). The P1NP/CTX-1 ratio was calculated to provide a quantitative measure of the bone formation ratio after treatments [45]. Here, the ratio increased at the T_2_ time point by 16.2% compared to baseline levels, but the measures were affected by great variability, and the result was not statistically significant (*p* = 0.95). Measurements on the reference BTM showed that 12 days of sulfurous treatment were not sufficient to induce an activation of bone metabolism.

Based on our previous data of a direct effect of sulfurous water in promoting mineral apposition by osteoblast precursors [46], we evaluated another BTM specifically related to mineralization by osteoblasts, bone-specific alkaline phosphatase (BALP). Notably, BALP was significantly increased after treatment at T_1_, achieving an 8% increase relative to baseline (*p* = 0.002); and the effect remained stable at T_2_ (*p* = 0.002). Controlling for age and BMI did not affect the result of this analysis. In addition to the graphical panels presented in Figure 3, Figure 4, Figure 5 and Figure 6, all biochemical measurements are summarized in Supplementary Table S1.

## 3. Discussion

This exploratory clinical trial provides the first evidence that the inhalation of sulfurous mineral waters enriched in H_2_S is associated with changes in systemic biomarkers of oxidative stress, inflammation, and mineralization in postmenopausal women with osteopenia. The treatment was well accepted and tolerated by patients, showing potential to achieve good adherence in the future.

Our findings are consistent with the hypothesis that sulfurous water can be used as natural H_2_S donor to increase H_2_S circulating levels and that exogenous H_2_S supplementation exerts antioxidant and anti-inflammatory effects in humans, complementing prior preclinical studies that have demonstrated protective roles of H_2_S in bone biology [47,48].

Previous work from our group found that, in vitro, the use of sulfurous water stimulates the osteogenic differentiation and mineralization of human mesenchymal stromal cells (MSCs) in a way that is similar to that of sulfide donors, and this effect is associated with an increased concentration of intracellular H_2_S [46]. Interestingly, in vivo studies revealed that a deficit in the level of circulating H_2_S is associated with the bone loss linked to ovariectomy [37] or to a sustained administration of glucocorticoids [49], suggesting that pathways implicated in the control of endogenous H_2_S may play an homeostatic role in bone metabolism. In both experimental models, the systemic administration of H_2_S donors reversed the bone loss and prevented the onset of osteoporosis [37,49]. While these data provide biological plausibility, extrapolation to the present clinical findings requires caution.

Notably, in our study, H_2_S levels measured in the serum of a subset of patients increased following inhalation and further increased (becoming statistically significant) at follow-up (T_2_), confirming the systemic bioavailability of H_2_S after the inhalation of sulfurous waters. This finding provides a link between the inhalation intervention and the observed biological effects on oxidative stress and inflammation. Importantly, no early adverse effects were reported, confirming the safety and tolerability of this non-pharmacological intervention approach. Our findings show higher H_2_S levels at T_2_ than at T_1_; we can speculate that the protein-bound form of H_2_S, which is likely the most stable one, may undergo a concentration buildup based on a longer half-life; further investigation should be directed to better understand the metabolic direction of H_2_S upon inhalation.

A major finding of this study is the significant and sustained reduction in oxidative stress following sulfurous water inhalation, as indicated by declining serum MDA levels. Oxidative stress has been consistently identified as an independent risk factor for osteoporosis, with excess ROS impairing osteoblast differentiation and survival while promoting osteoclast activity. Previous clinical studies have correlated higher levels of oxidative biomarkers such as AOPPs, MDA, and 8-OH-dG with low bone mineral density and fracture risk in postmenopausal women. More specifically, MDA was identified as playing a pathogenetic role in age-related disorders [50].

Our data show that a 12-day course of H_2_S inhalation substantially reduced circulating MDA levels, suggesting that targeting oxidative stress through H_2_S delivery may be a feasible non-pharmacological approach to preserve bone health in osteopenic women. Interestingly, a previous study performed on healthy patients undergoing hydropinic treatment with sulfurous waters, with a sulfuric content similar to ours (14.5 mg/L), showed a similar reduction in MDA levels [51]. Taken together, this evidence suggests that H_2_S-rich waters downregulate oxidative stress regardless of the way it is administered. Interestingly, it been reported that NRF2 regulates MDA levels [52,53] and we previously showed in vitro that H_2_S upregulates NRF2 in OC [39]; therefore, it may be speculated that sulfurous water inhalation reduces MDA levels through increased NRF2.

Another key result is the observed modulation of circulating inflammatory cytokines. Postmenopausal estrogen deficiency is associated with low-grade inflammation and the increased secretion of pro-resorptive cytokines such as IL-8 and MIP-1α [54,55], which accelerate osteoclastogenesis and bone resorption. In our cohort, the levels of IL-8 and MIP-1α were significantly lower after H_2_S inhalation, consistent with the known immunomodulatory properties of H_2_S. Notably, MIP-1α levels were found to negatively correlate with total bone BMD [56], highlighting a potential causality between this cytokine and BMD. Moreover, the effect observed on IL-8 in this study is coherent with preclinical studies published elsewhere [57] showing that H_2_S exerts a specific transcriptional downregulation of IL-8 both in vitro and in vivo. These findings highlight the potential of H_2_S to attenuate inflammation-driven bone loss and reinforce its role as a regulator of immune–bone interactions.

Regarding bone metabolism, we analyzed BTMs as useful biomarkers able to identify conditions or treatments affecting bone turnover [58]. Here we found that the inhalation treatment did not significantly alter bone turnover. We observed a modest and not statistically significant increase in BTMs (P1NP and CTX-1) immediately after treatment, followed by normalization at five days post-treatment. Interestingly, despite the short regimen of administration, we found a significant increase in another prototypical biomarker of bone anabolism, BALP. BALP is an isoform of alkaline phosphatase (ALP) found in the bone tissue, which plays a prominent role in the calcification process by increasing local concentrations of inorganic phosphate, degrading the mineralization inhibitor pyrophosphate, and acting as a calcium-binding protein [59]. Although BALP is not the reference standard for monitoring bone formation in the treatment of osteoporosis, its slow clearance and low intra-individual variability make it an appropriate marker of bone biomineralization [60]. Its significant increase is suggestive of an activation of biomineralization during the inhalation intervention and at least partially confirms our preliminary in vitro evidence of the effect of sulfurous water on the mineralization of MSCs [46].

While the variability in BTMs limits definitive interpretation, these exploratory findings justify further investigation in adequately powered randomized controlled trials to determine whether repeated or prolonged H_2_S inhalation cycles would translate into a measurable increase in BTMs of bone turnover, gains in bone density and reduced fracture risk.

Our study has several limitations. The relatively small sample size (n = 38), the lack of a control group and the short duration of the intervention weaken the interpretation of results and limit their generalizability. Furthermore, although we demonstrated biomarker changes, the method of administration did not allow us to assess long-term clinical endpoints such as BMD, fracture incidence, or sustained metabolic effects. Additional studies with larger randomized cohorts, including untreated groups, longer interventions, and functional outcomes, are of paramount importance to confirm the potential of H_2_S-based strategies to improve bone health.

Despite the limitations in study design, the present findings are significant for several reasons. First, they bridge a gap between preclinical research on H_2_S and translational application in human subjects; second, they demonstrate that sulfurous water inhalation could find clinical application in pathologies involving inflammatory and pro-oxidant processes other than the treatment of airway diseases. Most importantly, they provide the first preliminary evidence that H_2_S inhalation can influence multiple systemic pathways implicated in bone fragility; indeed, the coordinated changes in oxidative and inflammatory biomarkers, the magnitude of their increase, together with the documented rise in circulating H_2_S, indicate a biologically coherent treatment effect rather than random fluctuation. Overall, they suggest that mineral waters enriched in H_2_S, a widely accessible and well-tolerated therapy, have the potential to serve as a complementary non-pharmacological strategy to support bone health in postmenopausal women at risk of osteoporosis.

## 4. Materials and Methods

### 4.1. Ethics Statements

The protocol was approved by the Ethical Committee at IRCCS Istituto Ortopedico Rizzoli (IOR; Bologna, Italy), CE AVEC 625/2019/Sper/IOR, and registered at www.clinicaltrial.gov (Identifier NCT06095349). A preliminary screening of potential participants was conducted through phone interviews with women who had expressed interest in joining the study. During the interview, participants were informed about the study objectives and participation procedures and were assessed for inclusion and exclusion criteria. They were asked about their availability to attend a 12-day inhalatory treatment program at the Castel San Pietro Terme thermal center (Bologna, Italy).

### 4.2. Study Population and Design

This exploratory, single-arm trial was performed in two centers, respectively responsible for different activities: (1) IOR was responsible for enrollment, blood harvesting at T_0_ and T_2_, and experimental and data analysis; and the Castel San Pietro Terme thermal center was responsible for sulfurous water inhalation (as an accredited center for sulfurous thermal water inhalation) and for blood harvesting at T_1_. The rationale of the study design was based on enrolling women showing BMD in the range of osteopenia, as a key risk factor for developing osteoporosis, and the population with higher rates of fractures was an ideal target group for interventions supporting bone health.

Participants were consecutively enrolled at IOR. Inclusion criteria were as follows: postmenopausal status, age between 50 and 60 years, osteopenia defined as lumbar spine and/or femoral neck, BMD T score between −1.0 and −2.5 as compared to that of the reference population. A DXA scan was performed at IOR for all women who had not performed a DXA scan on their own within the last year before enrollment. Women were considered to have menopause if they were ≥40 years old and had no menstrual cycles for at least 1 year. Exclusion criteria for all participants were the presence of osteoporosis (defined by DXA analysis); any condition that can interfere with bone and calcium metabolism such as chronic kidney disease, metabolic bone diseases, inflammatory bowel diseases, rheumatic diseases, musculoskeletal disorders; and the use of medications such as bisphosphonates, teriparatide, corticosteroids, and aromatase inhibitors. Moreover, our cohort of women were not under hormone therapy with estrogen or similar (the most effective treatment for postmenopausal women with menopausal symptoms), since it could interfere both with bone metabolism as well as H_2_S production.

One-hundred-and-eight postmenopausal women were potentially eligible for the study and were screened against the inclusion criteria of the clinical protocol after providing informed consent: particularly, BMD was assessed by DXA scan on each woman to identify participants with BMD in the range of osteopenia.

Based on these criteria, seventy-two women were excluded from the study, mainly because they did not meet the inclusion criteria of a BMD in the range of osteopenia. As a result, the study enrolled thirty-eight postmenopausal, osteopenic women.

### 4.3. Treatment

Sulfurous water inhalation was performed at the center of Castel San Pietro Terme according to a standard clinical protocol used for rhinogenic deafness, respiratory and broncho-pulmonological diseases (pathologies for which inhalation treatments can be prescribed in agreement with the Italian National Health System, SSN). Specifically, eligible women underwent two consecutive cycles of 15 min for 12 days: the first was an inhalation with nebulized sulfurous waters, the second was the so-called ‘humage’, a dry inhalation treatment obtained with a special device that promotes the conversion of dissolved H_2_S in water into gaseous form, increasing the total amount of H_2_S inhaled. As reported in Table 1, the water used in this study has a sulfuric content of 14.6 mg/L.

Effects of inhalation were evaluated at three different time points: before treatment (T_0_), immediately after the end of the 12-day treatment (T_1_) and 5 days after the cessation of the treatment (T_2_). Biomarkers of oxidative stress, inflammation, bone turnover and H_2_S levels were measured across three time points.

Relevant demographics, BMD T scores, clinical characteristics and routine blood test results for the study participants are summarized in Table 2. All eligible women were in good health as determined by medical history questionnaires, routine blood tests and physical examinations.

### 4.4. DXA Scan

DXA scans (proximal femur and lumbar spine) were acquired at our institution by expert radiology technicians and analyzed by an expert musculoskeletal radiologist (P.S.). DXA equipment: Hologic, Discovery Wi (S/N86488), Marlborough, MA, USA.

### 4.5. Blood Sampling

At each time point, venous blood samples were collected from each subject using Vacutainer tubes. To mitigate for potential circadian variation in the levels of markers (especially CTX-1) blood was taken consistently between 12 and 2 p.m. at all time points. This time slot was chosen because the inhalation treatments were only available during the morning time, and we set blood harvesting at T_1_ as soon as the patients completed their treatment.

All parameters belonging to normal clinical practice were assayed on fresh blood samples at the Metropolitan Laboratory of the Bologna area (LUM). Specifically, the determination of creatinine, albumin, ALP, and calcium were performed by using the colorimetric kit from Beckman Coulter (Beckman Coulter, Brea, CA, USA); phosphate by using the photometric UV kit from Beckman Coulter; VITD and PTH by using the chemiluminescence kit from Beckman Coulter; CTX-1 and BALP by using, respectively, the chemiluminescence and spectrophotometric technology kits from IDS (Boldon, UK). Assessment of P1NP, MDA, and inflammatory cytokines were performed by means of laboratory assays and commercial kits on serum (as detailed below). Serum aliquots were obtained by blood samples centrifuged within 2 h from collection at 2500 rpm for 10 min at room temperature and then stored at −80 °C until assayed.

### 4.6. H_2_S Measurements

To investigate whether the sulfurous water inhalation increased the circulating levels of H_2_S, we measured serum levels of H_2_S in a subgroup of 4 patients randomly selected from enrolled participants; the demographic features of the subgroup of patients were representative of the entire population both for age (55 yrs vs. 54.9 yrs in the entire population) and for the years after menopause (4.4 yrs vs. 4.3 yrs in the entire population); measurement was performed following an optimized Monobromobimane derivatization protocol established in a previous work from our group (linearity 0.5 µM; limit of quantification 0.9 µM) [44], and using an Agilent 1260 Infinity HPLC system (Santa Clara, CA, USA), equipped with a G1379B degasser, G1312B binary gradient pump, G1329B autosampler, G4212B diode array detector, and G1321A fluorescence detector.

### 4.7. Biochemical Measurements

Serum cytokine levels were monitored during the study by using a fluorescent-labeled microsphere-based multiplex immunoassay (Bio-Plex Pro Human Cytokine 27-plex assay, Biorad, Segrate (Milan), Italy) according to the manufacturer’s instructions. The intra- and inter-assay coefficients of variation for the detectable cytokines were as follows: G-CSF 3.1–4%; IL-4 3.2–1.9%; IL-8 3.2–2.8%; IL-9 2.6–7.1%; IP-10 2.8–6%; MCP-1 3.2–3.4%; MIP-1α 4.5–4.2%; MIP-1β 3.4–2.5%; platelet-derived growth factor (PDGF-) 3.3–9.7%; TNF-α 3.5–3%.

Assessment of oxidative stress status was performed by measuring serum levels of MDA, the highly reactive byproduct of lipid metabolism which causes toxic stress and forms covalent protein adducts, using the MDA colorimetric kit, with a sensitivity of 18.75 ng/mL and analytical range of 31.25–2000 ng/mL (Invitrogen, Thermo Fisher Scientific, Waltham, MA, USA), following the manufacturer’s instructions. The intra-assay CV and inter-assay CV were <10%.

Determination of P1NP was performed by using a P1NP ELISA kit (My Biosource, code MBS398102, San Diego, CA, USA), with a sensitivity of 62.5 pg/mL, following the manufacturer’s instructions. The intra-assay CV and inter-assay CV were, respectively, <3.1% and <5.1%. All biochemical measurements are summarized in Supplementary Table S1.

### 4.8. Statistical Analysis

Statistical analyses were performed using SPSS version 23 (IBM SPSS Statistics, Chicago, IL, USA). Continuous variables are presented as mean ± standard deviation (SD) or median and interquartile range (IQR), as appropriate according to data distribution, while categorical variables are expressed as absolute numbers and percentages. Normality of distribution was assessed using the Kolmogorov–Smirnov and Shapiro–Wilk tests. Generalized linear mixed models (GLMMs) with a lognormal distribution were applied to evaluate the longitudinal variation in the investigated parameters (e.g., MDA, IL-8, and other biomarkers) over time. Time was included as a fixed effect, while patients were modeled as a random effect to account for within-subject correlation. Potential confounders (e.g., age, BMI) were included as covariates within the models. Estimated regression coefficients are presented with their 95% confidence intervals (CIs). When appropriate, post hoc pairwise comparisons were performed, and *p*-values were adjusted for multiple testing using the false discovery rate (FDR) correction. All statistical tests were two-sided, and *p*-values < 0.05 were considered statistically significant.

## 5. Conclusions

Based on the preclinical evidence supporting a role of H_2_S in preventing osteoporosis in animal models, this exploratory study evaluated the feasibility of sulfurous water inhalation in osteopenic women and the associated biomarker profile. Our findings showed that a 12-day cycle of treatment modulated systemic pathways implicated in bone fragility by inducing a decrease in inflammatory markers (especially IL-8, MIP-1α) and antioxidant markers (MDA), showing that pathologies other than airway diseases can be modulated by sulfurous inhalation. In concordance with previous evidence from in vitro studies showing a direct stimulation of osteogenesis in human MSC by H_2_S-rich water, this study showed an increase in the systemic levels of the biomineralization marker BALP. Given the non-controlled design, these findings should be considered preliminary and hypothesis-generating. The results support the feasibility and short-term tolerability of this intervention and provide a rationale for larger clinical trials demonstrating the potential of sulfurous water inhalation as a complementary non-pharmacological strategy in postmenopausal women at risk of osteoporosis.

## Figures and Tables

**Table 1:** Chemical characteristics of the sulfurous mineral waters used in the study.

Parameter	Value	Unit
pH	7.5	n/a
NH_4_	1.7	mg/L
NO_2_	<0.01	mg/L
NO_3_	<1	mg/L
Cl^−^	106	mg/L
SO_4_^2−^	67	mg/L
HCO_3_	462	mg/L
Ca	126.1	mg/L
Mg	29.5	mg/L
Na	73.2	mg/L
K	4	mg/L
Li	0.101	mg/L
Sr	0.75	mg/L
Al	<0.004	mg/L
Fe soluble	<0.01	mg/L
SiO_2_	16.74	mg/L
H_2_S	14.6	mg/L

**Table 2:** Demographics, main clinical characteristics and serum biomarkers of the enrolled participants.

	Baseline	Post-Treatment
Age (years)	54.9 (±2.7)	54.9 (±2.7)
Body weight (kg)	65.5 (±10.6)	65.5 (±10.6)
BMI (kg/m^2^)	23.3 (±3.22)	23.3 (±3.22)
Femoral neck BMD (T score)	−1.4 (±0.23)	-
Lumbar BMD (L1-L4 T score)	−1.73 (±0.48)	-
Menopause (age)	50.4 (±2.6)	50.4 (±2.6)
Years after menopause (years)	4.3 (±3.1)	4.3 (±3.1)
Creatinine (mg/dL)	0.78 (±0.1)	0.77 (±0.1)
Albumin (mg/mL)	4.32 (±0.27)	4.35 (±0.27)
ALP (U/L)	73.11 (±2.69)	73.06 (±3.20)
Calcium (mg/dL)	9.84 (±0.43)	9.68 (±0.37)
Phosphate (mg/dL)	3.65 (±0.43)	3.71 (±0.46)

## Data Availability

The original contributions presented in this study are included in the article/Supplementary Material. Further inquiries can be directed to the corresponding author.

## Supplementary Materials

The following supporting information can be downloaded at: https://www.mdpi.com/article/10.3390/ijms27073163/s1.

## References

[1] Aibar-Almazan A., Voltes-Martinez A., Castellote-Caballero Y., Afanador-Restrepo D.F., Carcelen-Fraile M.D.C., Lopez-Ruiz E. (2022). Current Status of the Diagnosis and Management of Osteoporosis. Int. J. Mol. Sci..

[2] Kanis J.A. (1994). Assessment of fracture risk and its application to screening for postmenopausal osteoporosis: Synopsis of a WHO report. WHO Study Group. Osteoporos. Int..

[3] Dong Y., Kang H., Peng R., Song K., Guo Q., Guan H., Zhu M., Ye D., Li F. (2022). Global, Regional, and National Burden of Low Bone Mineral Density From 1990 to 2019: Results From the Global Burden of Disease Study 2019. Front. Endocrinol..

[4] Xiao P.L., Cui A.Y., Hsu C.J., Peng R., Jiang N., Xu X.H., Ma Y.G., Liu D., Lu H.D. (2022). Global, regional prevalence, and risk factors of osteoporosis according to the World Health Organization diagnostic criteria: A systematic review and meta-analysis. Osteoporos. Int..

[5] Lee K.I., Chen J.H., Chen K.H. (2026). Osteoporosis After Menopause and After Drug Therapy: The Molecular Mechanism of Bone Loss and Its Treatment. Int. J. Mol. Sci..

[6] Cauley J.A., Robbins J., Chen Z., Cummings S.R., Jackson R.D., LaCroix A.Z., LeBoff M., Lewis C.E., McGowan J., Neuner J. (2003). Effects of estrogen plus progestin on risk of fracture and bone mineral density: The Women’s Health Initiative randomized trial. JAMA.

[7] Khosla S., Monroe D.G. (2018). Regulation of Bone Metabolism by Sex Steroids. Cold Spring Harb. Perspect. Med..

[8] Khosla S., Melton L.J. (2007). Clinical practice. Osteopenia. N. Engl. J. Med..

[9] Gourlay M.L., Fine J.P., Preisser J.S., May R.C., Li C., Lui L.Y., Ransohoff D.F., Cauley J.A., Ensrud K.E., Study of Osteoporotic Fractures Research G. (2012). Bone-density testing interval and transition to osteoporosis in older women. N. Engl. J. Med..

[10] Reid I.R., McClung M.R. (2024). Osteopenia: A key target for fracture prevention. Lancet Diabetes Endocrinol..

[11] Baquero Ubeda J.L., Garcia Diaz S., Gomez Martinez J.C., Martinez Fernandez N., Mico Perez R.M., Moller I., Neyro Bilbao J.L., Saez Lopez P., Santina Vila M., Verges Milano J. (2025). Multidisciplinary consensus on the management of patients with osteopenia and fracture risk in Spain. BMC Musculoskelet. Disord..

[12] Mun J., Kim M., Song J., Chung Y., Park J., Park J. (2025). Individualized Fracture Prevention for Postmenopausal Women with Osteopenia. J. Menopausal Med..

[13] Cenci S., Weitzmann M.N., Roggia C., Namba N., Novack D., Woodring J., Pacifici R. (2000). Estrogen deficiency induces bone loss by enhancing T-cell production of TNF-alpha. J. Clin. Investig..

[14] Cline-Smith A., Axelbaum A., Shashkova E., Chakraborty M., Sanford J., Panesar P., Peterson M., Cox L., Baldan A., Veis D. (2020). Ovariectomy Activates Chronic Low-Grade Inflammation Mediated by Memory T Cells, Which Promotes Osteoporosis in Mice. J. Bone Miner. Res..

[15] Iantomasi T., Romagnoli C., Palmini G., Donati S., Falsetti I., Miglietta F., Aurilia C., Marini F., Giusti F., Brandi M.L. (2023). Oxidative Stress and Inflammation in Osteoporosis: Molecular Mechanisms Involved and the Relationship with microRNAs. Int. J. Mol. Sci..

[16] Livshits G., Kalinkovich A. (2022). Targeting chronic inflammation as a potential adjuvant therapy for osteoporosis. Life Sci..

[17] El Khoudary S.R., Chen X., Qi M., Matthews K.A., Karlamangla A., Gold E.B., Harlow S.D., Thurston R.C., Joffe H., Pavlovic J. (2025). The Relation between Systemic Inflammation and the Menopause Transition: The Study of Women’s Health Across the Nation. J. Clin. Endocrinol. Metab..

[18] Altindag O., Erel O., Soran N., Celik H., Selek S. (2008). Total oxidative/anti-oxidative status and relation to bone mineral density in osteoporosis. Rheumatol. Int..

[19] Grassi F., Tell G., Robbie-Ryan M., Gao Y., Terauchi M., Yang X., Romanello M., Jones D.P., Weitzmann M.N., Pacifici R. (2007). Oxidative stress causes bone loss in estrogen-deficient mice through enhanced bone marrow dendritic cell activation. Proc. Natl. Acad. Sci. USA.

[20] Luo J., Li L., Shi W., Xu K., Shen Y., Dai B. (2025). Oxidative stress and inflammation: Roles in osteoporosis. Front. Immunol..

[21] Kimball J.S., Johnson J.P., Carlson D.A. (2021). Oxidative Stress and Osteoporosis. J. Bone Joint. Surg. Am..

[22] Wu Q., Zhong Z.M., Pan Y., Zeng J.H., Zheng S., Zhu S.Y., Chen J.T. (2015). Advanced Oxidation Protein Products as a Novel Marker of Oxidative Stress in Postmenopausal Osteoporosis. Med. Sci. Monit..

[23] Mundy G.R. (2007). Osteoporosis and inflammation. Nutr. Rev..

[24] Baek K.H., Oh K.W., Lee W.Y., Lee S.S., Kim M.K., Kwon H.S., Rhee E.J., Han J.H., Song K.H., Cha B.Y. (2010). Association of oxidative stress with postmenopausal osteoporosis and the effects of hydrogen peroxide on osteoclast formation in human bone marrow cell cultures. Calcif. Tissue Int..

[25] Lee N.K., Choi Y.G., Baik J.Y., Han S.Y., Jeong D.W., Bae Y.S., Kim N., Lee S.Y. (2005). A crucial role for reactive oxygen species in RANKL-induced osteoclast differentiation. Blood.

[26] Tao H., Li X., Wang Q., Yu L., Yang P., Chen W., Yang X., Zhou J., Geng D. (2024). Redox signaling and antioxidant defense in osteoclasts. Free Radic. Biol. Med..

[27] Bai X.C., Lu D., Bai J., Zheng H., Ke Z.Y., Li X.M., Luo S.Q. (2004). Oxidative stress inhibits osteoblastic differentiation of bone cells by ERK and NF-kappaB. Biochem. Biophys. Res. Commun..

[28] Fontani F., Marcucci G., Iantomasi T., Brandi M.L., Vincenzini M.T. (2015). Glutathione, N-acetylcysteine and lipoic acid down-regulate starvation-induced apoptosis, RANKL/OPG ratio and sclerostin in osteocytes: Involvement of JNK and ERK1/2 signalling. Calcif. Tissue Int..

[29] Sbodio J.I., Snyder S.H., Paul B.D. (2019). Regulators of the transsulfuration pathway. Br. J. Pharmacol..

[30] Flori L., Veneziano S., Martelli A., Piragine E., Calderone V. (2025). Transsulfuration Pathway Products and H_2_S-Donors in Hyperhomocysteinemia: Potential Strategies Beyond Folic Acid. Int. J. Mol. Sci..

[31] Paul B.D., Snyder S.H. (2015). H2S: A Novel Gasotransmitter that Signals by Sulfhydration. Trends Biochem. Sci..

[32] Vandiver M., Snyder S.H. (2012). Hydrogen sulfide: A gasotransmitter of clinical relevance. J. Mol. Med..

[33] Wang R. (2012). Physiological implications of hydrogen sulfide: A whiff exploration that blossomed. Physiol. Rev..

[34] Cirino G., Szabo C., Papapetropoulos A. (2023). Physiological Roles of Hydrogen Sulfide in Mammalian Cells, Tissues, and Organs. Physiol. Rev..

[35] Liu Y., Yang R., Liu X., Zhou Y., Qu C., Kikuiri T., Wang S., Zandi E., Du J., Ambudkar I.S. (2014). Hydrogen sulfide maintains mesenchymal stem cell function and bone homeostasis via regulation of Ca^2+^ channel sulfhydration. Cell Stem Cell.

[36] Xu Z.S., Wang X.Y., Xiao D.M., Hu L.F., Lu M., Wu Z.Y., Bian J.S. (2011). Hydrogen sulfide protects MC3T3-E1 osteoblastic cells against H2O2-induced oxidative damage-implications for the treatment of osteoporosis. Free Radic. Biol. Med..

[37] Grassi F., Tyagi A.M., Calvert J.W., Gambari L., Walker L.D., Yu M., Robinson J., Li J.Y., Lisignoli G., Vaccaro C. (2016). Hydrogen Sulfide Is a Novel Regulator of Bone Formation Implicated in the Bone Loss Induced by Estrogen Deficiency. J. Bone Miner. Res..

[38] Behera J., Kelly K.E., Voor M.J., Metreveli N., Tyagi S.C., Tyagi N. (2018). Hydrogen Sulfide Promotes Bone Homeostasis by Balancing Inflammatory Cytokine Signaling in CBS-Deficient Mice through an Epigenetic Mechanism. Sci. Rep..

[39] Gambari L., Lisignoli G., Cattini L., Manferdini C., Facchini A., Grassi F. (2014). Sodium hydrosulfide inhibits the differentiation of osteoclast progenitor cells via NRF2-dependent mechanism. Pharmacol. Res..

[40] Lee S.K., Chung J.H., Choi S.C., Auh Q.S., Lee Y.M., Lee S.I., Kim E.C. (2013). Sodium hydrogen sulfide inhibits nicotine and lipopolysaccharide-induced osteoclastic differentiation and reversed osteoblastic differentiation in human periodontal ligament cells. J. Cell. Biochem..

[41] Carubbi C., Masselli E., Calabro E., Bonati E., Galeone C., Andreoli R., Goldoni M., Corradi M., Sverzellati N., Pozzi G. (2019). Sulphurous thermal water inhalation impacts respiratory metabolic parameters in heavy smokers. Int. J. Biometeorol..

[42] Galvez I., Torres-Piles S., Ortega-Rincon E. (2018). Balneotherapy, Immune System, and Stress Response: A Hormetic Strategy?. Int. J. Mol. Sci..

[43] Contoli M., Gnesini G., Forini G., Marku B., Pauletti A., Padovani A., Casolari P., Taurino L., Ferraro A., Chicca M. (2013). Reducing agents decrease the oxidative burst and improve clinical outcomes in COPD patients: A randomised controlled trial on the effects of sulphurous thermal water inhalation. Sci. World J..

[44] Roda B., Zhang N., Gambari L., Grigolo B., Eller-Vainicher C., Gennari L., Zappi A., Giordani S., Marassi V., Zattoni A. (2022). Optimization of a Monobromobimane (MBB) Derivatization and RP-HPLC-FLD Detection Method for Sulfur Species Measurement in Human Serum after Sulfur Inhalation Treatment. Antioxidants.

[45] Fisher A., Srikusalanukul W., Fisher L., Smith P.N. (2017). Lower serum P1NP/betaCTX ratio and hypoalbuminemia are independently associated with osteoporotic nonvertebral fractures in older adults. Clin. Interv. Aging.

[46] Gambari L., Grigolo B., Filardo G., Grassi F. (2020). Sulfurous thermal waters stimulate the osteogenic differentiation of human mesenchymal stromal cells—An in vitro study. Biomed. Pharmacother..

[47] Liu Y.F., Zhang Y.X., Zhu Y.W., Tang A.Q., Liang H.B., Yang Y.L., Zhai Y.K., Ji X.Y., Wu D.D. (2025). Hydrogen Sulfide in Musculoskeletal Diseases: Molecular Mechanisms and Therapeutic Opportunities. Antioxid. Redox Signal..

[48] Hao Y., Wang H., Fang L., Bian J., Gao Y., Li C. (2021). H2S Donor and Bone Metabolism. Front. Pharmacol..

[49] Ma J., Shi C., Liu Z., Han B., Guo L., Zhu L., Ye T. (2019). Hydrogen sulfide is a novel regulator implicated in glucocorticoids-inhibited bone formation. Aging.

[50] Barrera G., Pizzimenti S., Daga M., Dianzani C., Arcaro A., Cetrangolo G.P., Giordano G., Cucci M.A., Graf M., Gentile F. (2018). Lipid Peroxidation-Derived Aldehydes, 4-Hydroxynonenal and Malondialdehyde in Aging-Related Disorders. Antioxidants.

[51] Benedetti S., Benvenuti F., Nappi G., Fortunati N.A., Marino L., Aureli T., De Luca S., Pagliarani S., Canestrari F. (2009). Antioxidative effects of sulfurous mineral water: Protection against lipid and protein oxidation. Eur. J. Clin. Nutr..

[52] Li H., Wang F., Zhang L., Cao Y., Liu W., Hao J., Liu Q., Duan H. (2011). Modulation of Nrf2 expression alters high glucose-induced oxidative stress and antioxidant gene expression in mouse mesangial cells. Cell Signal..

[53] Dodson M., Castro-Portuguez R., Zhang D.D. (2019). NRF2 plays a critical role in mitigating lipid peroxidation and ferroptosis. Redox Biol..

[54] Bendre M.S., Montague D.C., Peery T., Akel N.S., Gaddy D., Suva L.J. (2003). Interleukin-8 stimulation of osteoclastogenesis and bone resorption is a mechanism for the increased osteolysis of metastatic bone disease. Bone.

[55] Tsubaki M., Kato C., Isono A., Kaneko J., Isozaki M., Satou T., Itoh T., Kidera Y., Tanimori Y., Yanae M. (2010). Macrophage inflammatory protein-1alpha induces osteoclast formation by activation of the MEK/ERK/c-Fos pathway and inhibition of the p38MAPK/IRF-3/IFN-beta pathway. J. Cell. Biochem..

[56] Xu T., Li C., Liao Y., Zhang X. (2024). Causal relationship between circulating levels of cytokines and bone mineral density: A mendelian randomization study. Cytokine.

[57] Mirandola P., Gobbi G., Micheloni C., Vaccarezza M., Di Marcantonio D., Ruscitti F., de Panfilis G., Vitale M. (2011). Hydrogen sulfide inhibits IL-8 expression in human keratinocytes via MAP kinase signaling. Lab. Investig..

[58] Jain S., Camacho P. (2018). Use of bone turnover markers in the management of osteoporosis. Curr. Opin. Endocrinol. Diabetes Obes..

[59] Ponzano M., Wiest M.J., Coleman A., Newton E., Pakosh M., Patsakos E.M., Magnuson D.S.K., Giangregorio L.M., Craven B.C. (2023). The use of alkaline phosphatase as a bone turnover marker after spinal cord injury: A scoping review of human and animal studies. J. Spinal Cord Med..

[60] Brown J.P., Albert C., Nassar B.A., Adachi J.D., Cole D., Davison K.S., Dooley K.C., Don-Wauchope A., Douville P., Hanley D.A. (2009). Bone turnover markers in the management of postmenopausal osteoporosis. Clin. Biochem..

