# Supplementary material for: Changes in Oxidative Stress, Inflammatory and Bone Metabolism Biomarkers Following Sulfurous Water Inhalation in Osteopenic Women
Source: Int J Mol Sci. 2026 Mar 31;27(7):3163. doi: 10.3390/ijms27073163 (PMC13072851; doi:10.3390/ijms27073163)
Supplement: Supplementary file 1 [file ijms-27-03163-s001.zip › ijms-4201873-supplementary.pdf]

	T_0 (Mean, 95%CI)	T_1 (Mean, 95%CI)	T_2 (Mean, 95%CI)	P value	Post-hoc pairwise comparison	
					**T_0-T_1**	**T_0-T_2**
MDA	10.48 (*8.54, 12.86*)	8.61 (*6.86, 10.80*)	6.57 (*5.13, 8.41*)	p<0.0005	*p=0.048*	*p<0.001*
IL-8	82.86 (*57.26, 119.9*)	20.42 (*10.18, 40.95*)	16.20 (*6.99, 37.54*)	p<0.0005	*p<0.001*	*p<0.001*
MCP-1	34.35 (*29.28, 40.29*)	32.34 (*27.47, 38.08*)	31.65 (*26.82, 37.34)*	p=0.68	*n/a*	*n/a*
TNF-α	61.97 (*56.59, 67.36*)	61.09 (*55.24, 66.93)*	58.84 (*54.26, 63.41)*	p=0.68	*n/a*	*n/a*
IP-10	113.68 (*92.04, 135.3*)	99.12 (*82.31, 115.9*)	104.05 (*85.9, 122.19*)	P=0.62	*n/a*	*n/a*
IL-4	20.7 (*18.1, 23.4*)	22.3 (*17.06, 27.6*)	20.65 (*18, 23.3*)	P=0.37	*n/a*	*n/a*
IL-9	198 (*189.8, 206.3*)	201.5 (*193.4, 209.6*)	204.4 (*194.2, 214.6*)	P=0.26	*n/a*	*n/a*
MIP1-α	106 (*50.1, 161.8*)	56.5 (*38.1, 74.8*)	33.9 (*21.45, 46.4*)	p = 0.02	*p=0.09*	*P<0.01*
MIP1-β	787 (748.6, *825.4*)	793 (759.3, *826.7*)	814.1 (776.6, 851.6)	p=0.25	*n/a*	*n/a*
G-CSF	291 (*192.6, 339.4*)	225.9 (*114.5, 337.3*)	133.6 (*20.6, 246.5*)	p=0.109	*n/a*	*n/a*
PDGF	68.9 (*56.1, 84.6*)	56.4 (*45.8, 69.6*)	61.55 (*49.8, 76*)	p=0.22	*n/a*	*n/a*
1-25 VITD OH	28.73 (26.38, *31.28*)	29.52 (*27.11, 32.14*)	29.84 (*27.33, 32.57*)	p=0.26	*n/a*	*n/a*
PTH	47.01 (*41.42, 53.36*)	46.99 (*41.10, 53.72*)	47.47 (*41.77, 53.96*)	p=0.92	*n/a*	*n/a*
P1NP	14.62 (*12.98, 16.45*)	15.67 (*13.86, 17.72*)	14.95 (*13.25, 16.88*)	p=0.62	*n/a*	*n/a*
CTX-1	0.252 (*0.206, 0.308*)	0.267 (*0.217, 0.329*)	0.254 (*0.207, 0.311*)	p=0.78	*n/a*	*n/a*
BALP	15.12 (*13.76, 16.61*)	16.32 (*14.85, 17.94*)	16.27 (*14.81, 17.87*)	p=0.001	*p=0.002*	*p=0.002*

Supplementary Table S1. Tabular expression of serum levels of bone turnover markers, biomarkers of oxidative stress, inflammation, hormonal regulation of bone metabolism. T0 = baseline; T1 = end of treatment; T2 = 5 days follow-up after end of treatment.
